# Brain-derived neurotrophic factor, a new soluble biomarker for malignant pleural mesothelioma involved in angiogenesis

**DOI:** 10.1186/s12943-018-0891-0

**Published:** 2018-10-11

**Authors:** Patrick Smeele, Sènan Mickaël d’Almeida, Clément Meiller, Anne-Laure Chéné, Charly Liddell, Laurent Cellerin, François Montagne, Sophie Deshayes, Sarah Benziane, Marie-Christine Copin, Paul Hofman, Françoise Le Pimpec-Barthes, Henri Porte, Arnaud Scherpereel, Marc Grégoire, Didier Jean, Christophe Blanquart

**Affiliations:** 1 CRCINA, INSERM, Université d’Angers, Université de Nantes, Nantes, France; 2 Massachusetts General Hospital, Harvard Medical School, Boston, USA; 3 INSERM, UMR-1162, Functional Genomics of Solid Tumors, Université Paris Descartes, Université Paris Diderot, Université Paris 13, Paris, France; 4 Service d’Oncologie Médicale Thoracique et Digestive, Hôpital Laënnec, CHU de Nantes, Nantes, France; 5 Service d’Anatomie Pathologique, Hôpital Laënnec, CHU de Nantes, Nantes, France; 6 Pulmonary and Thoracic Oncology, CHU de Lille, Univ. Lille, INSERM U1019, CIIL Institut Pasteur de Lille, F59000 Lille, France; 7 French National Network of Clinical Expert Centers for Malignant Pleural Mesothelioma Management (MESOCLIN), F59000 Lille, France; 8 Univ. Lille, CHU Lille, Institut de Pathologie et Tumorothèque du C2RC, Avenue Oscar Lambret, F-59000 Lille, France; 9 Laboratory of Clinical and Experimental Pathology and Hospital-related Biobank (BB-0033-00025), University Côte d’Azur, Nice, France; 10 Département de Chirurgie Thoracique et Transplantation pulmonaire, Hôpital Européen Georges Pompidou, Paris, France; 11 Service de Chirurgie Thoracique, Hôpital Calmette, CHRU Lille, Lille, France

**Keywords:** BDNF, Mesothelioma, Pleural effusions, Biomarkers, Angiogenesis

## Abstract

Malignant pleural mesothelioma (MPM) is a rare and aggressive cancer related to asbestos exposure. The discovery of soluble biomarkers with diagnostic/prognostic and/or therapeutic properties would improve therapeutic care of MPM patients. Currently, soluble biomarkers described present weaknesses preventing their use in clinic. This study aimed at evaluating brain-derived neurotrophic factor (BDNF), we previously identified using transcriptomic approach, in MPM. We observed that high BDNF expression, at the mRNA level in tumors or at the protein level in pleural effusions (PE), was a specific hallmark of MPM samples. This protein presented significant but limited diagnostic properties (area under the curve (AUC) = 0.6972, *p* < 0.0001). Interestingly, high *BDNF* gene expression and PE concentration were predictive of shorter MPM patient survival (13.0 vs 8.3 months, p < 0.0001, in PE). Finally, BDNF did not affect MPM cell oncogenic properties but was implicated in PE-induced angiogenesis. In conclusion, BDNF appears to be a new interesting biomarker for MPM and could also be a new therapeutic target regarding its implication in angiogenesis.

**Electronic supplementary material:**

The online version of this article (10.1186/s12943-018-0891-0) contains supplementary material, which is available to authorized users.

Malignant pleural mesothelioma (MPM) is a rare and aggressive cancer related to asbestos exposure. The first line regimen for MPM, consisting of a combination of cisplatin and the anti-metabolite pemetrexed, only increases patient survival by 3 months [1]. The late diagnosis of the disease is partly responsible for the poor outcome in MPM. Thus, the identification of new biomarkers with diagnostic/prognostic and/or therapeutic properties would be useful to improve the therapeutic care of patients and the outcome of the disease. Soluble biomarkers have the advantage of being easily measured in fluid samples without the need to resort to invasive procedures and also to be targetable using antibodies. Previously identified MPM soluble biomarkers, soluble mesothelin-related peptide (SMRP) and fibulin-3, are too limited to be used routinely in clinic and are not identified as therapeutic target [2]. Therefore, the identification of new soluble biomarkers with improved or complementary properties is required.

In a previous study, we identified BDNF, a neurotrophin, as an interesting biomarker for MPM [3]. In this work, we aimed at examining this potential using collections of MPM samples. We also studied the implication of BDNF in MPM pathology.

## Results and discussion

### BDNF mRNA expression in MPM tumors and prognostic value

**Fig. 1:**
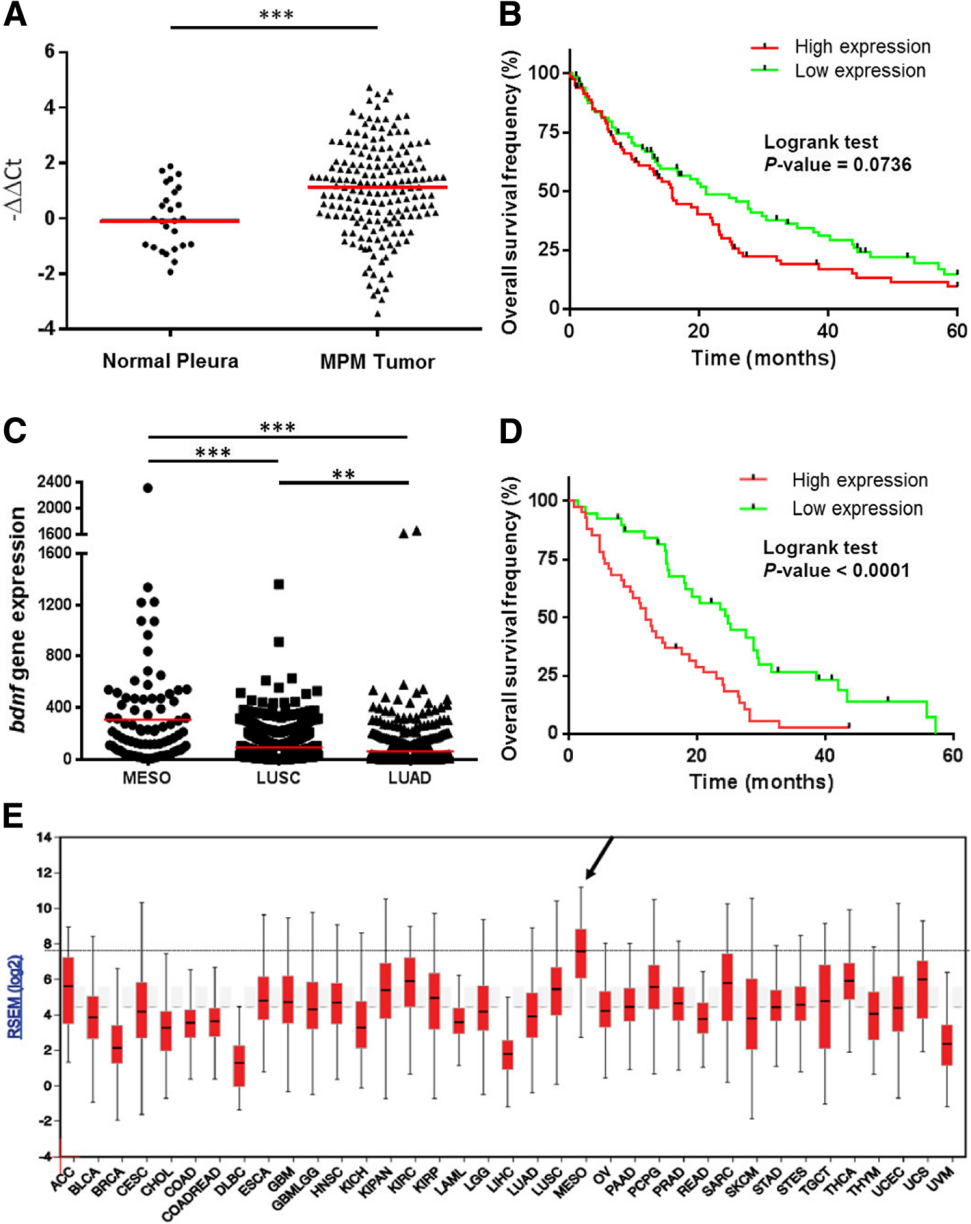
*BDNF* mRNA expression in MPM tumors and prognostic value. **a**, **b** Data from frozen MPM tumors samples collection **a** mRNA expression of *BDNF* in MPM tumors and normal pleura. Red bars correspond to median. ***p < 0.001. **b** Overall survival of MPM patients. Patients were separated in “high expression” and “low expression” groups based on the *BDNF* mRNA expression median and differences in survival between two groups are assessed by log-rank tests. **c**-**e**) Data from TCGA database. **c** mRNA expression of *BDNF* in MPM tumors, lung adenocarcinoma (LUAD) and lung squamous cell carcinoma (LUSC). Red bars correspond to median. ***p < 0.001. **d** Overall survival of MPM patients. Patients were separated in “high expression” and “low expression” groups based on the *BDNF* mRNA expression median and differences in survival between two groups are assessed by log-rank tests. **e** Expression of *BDNF* mRNA in 37 different cancers. Arrow indicates mesothelioma *BDNF* expression. Black horizontal line corresponds to median of *BDNF* mRNA expression in MPM samples

Previous transcriptomic data show an overexpression of *BDNF* gene expression in MPM cell lines compared to lung adenocarcinoma cell lines (Additional file 1: Figure S1) [3]. To confirm these results, *BDNF* expression was measured in 179 MPM tumor samples and 26 normal pleura (Additional file 2: Table S1.1). Figure 1a confirms the significant higher expression of *BDNF* in MPM tumors compared to normal pleura (*p* = 0.0006). *BDNF* showed differential expression between MPM subtypes (*p* = 0.0011) with a lower expression in epithelioid MPM (EM) than in sarcomatoid (SM) and desmoplastic (DM) MPM (Additional file 3: Figure S2A).

*BDNF* expression and overall survival of patients were related (Fig. 1b and Additional file 4: Table S2). Indeed, patients with high *BDNF* had a lower survival than patients with low *BDNF* (15.9 versus 21.1 months, *p* = 0.0736) and this survival difference is significant at 3 years (*p* = 0.0401).

These observations were confirmed using TCGA database (Additional file 2: Table S1.2). Expression of *BDNF* was significantly higher in MPM than in lung squamous carcinoma and lung adenocarcinoma (Fig. 1c). As previously observed, high *BDNF* was associated with low survival compared to low *BDNF* (12.4 versus 27.5 months, *p* < 0.0001) (Fig. 1d and Additional file 4: Table S2). *BDNF* was already described as overexpressed in several other cancers [4]. In TCGA cohort, we observed that MPM has the highest *BDNF* expression among 37 tumor types indicating that *BDNF* gene overexpression is a hallmark of MPM (Fig. 1e and Additional file 5: Table S3). These results were confirmed at the mRNA level and using Immunofluorescence on cancer cell lines and commercial primary mesothelial cells (MC) (Additional file 6: Figure S3A-B).

### Expression of BDNF in pleural effusions from patients

**Fig. 2:**
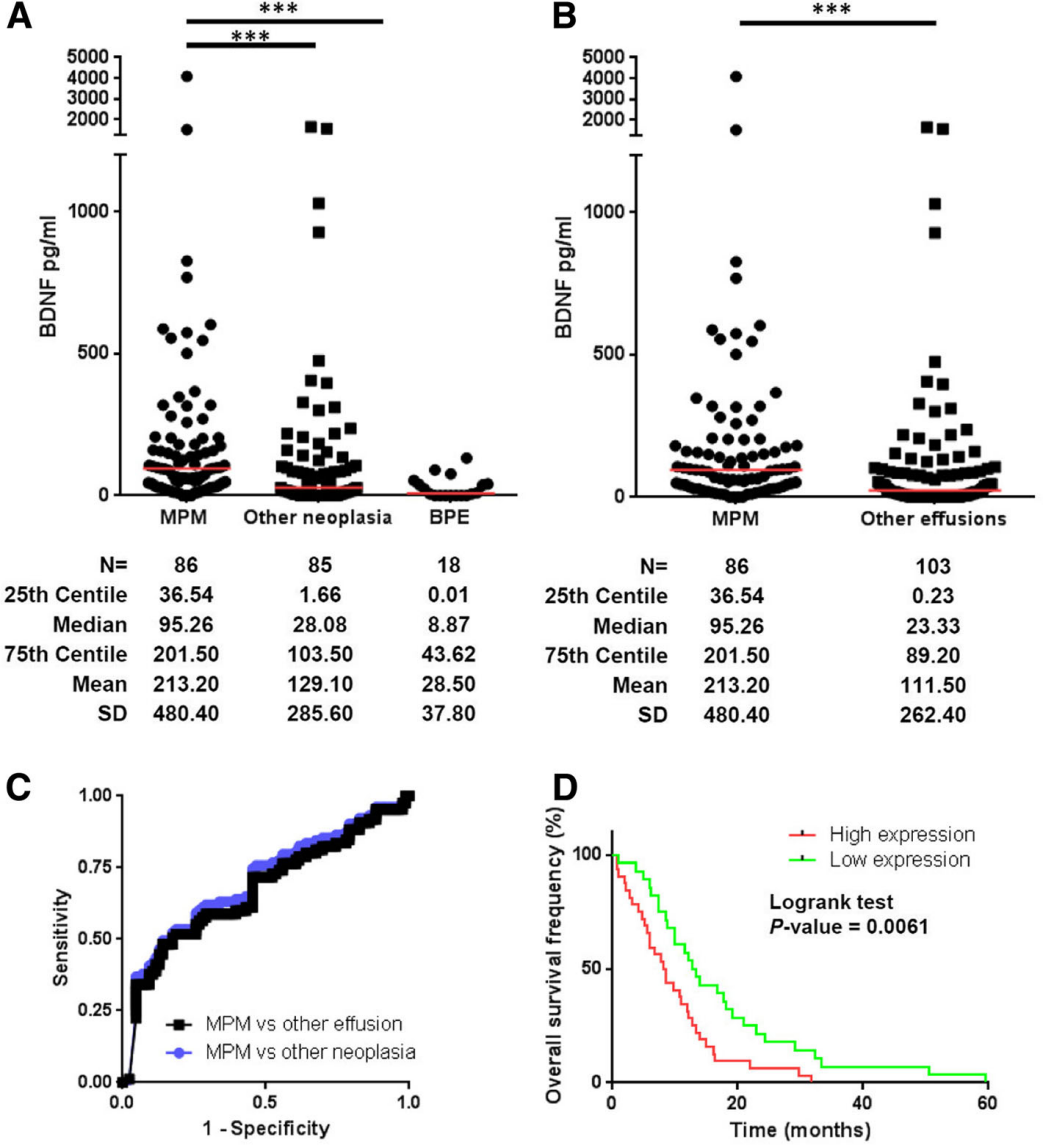
Diagnostic and prognostic value of BDNF in pleural effusions from patients. Pleural fluid BDNF values **a** in patients with MPM, other neoplasia or BPE or **b** in patients with MPM or other effusions (neoplasia and BPE). Red bars correspond to median. ***p < 0.001. MPM. malignant pleural mesothelioma; BPE. benign pleural effusion. **c** ROC curve for BDNF to distinguish between patients with MPM and patients with other malignant and/or benign effusions. **d** Overall Survival of MPM patients. Patients were separated in “high expression” and “low expression” groups based on the BDNF expression median in MPM PE and differences in survival between two groups are assessed by log-rank tests

In our collection of pleural effusions (PE) (Additional file 2: Table S1.3), a significant higher BDNF level was observed in MPM samples (median, 95.26 pg/ml) compared to other neoplasia or benign samples (BPE) (median, 28.08 pg/ml and 8.87 pg/ml) (Fig. 2a) and also to all PE (malignant and non-malignant) (median, 23.33 pg/ml) (Fig. 2b) according to the mRNA results. No significant difference in BDNF level was observed between the MPM subgroups (Additional file 3: Figure S2B).

These results confirmed a preliminary observation by Duysinx and colleagues performed on only 10 MPM PE [4] and can be explained, in part, by the ability of MPM cells to produce high level of BDNF (Additional file 6: Figure S3C). This growth factor can also be produced by a large variety of cells [5] explaining its presence in other PE, but at a lower amount.

Area under the curve (AUC) of BDNF to differentiate MPM from other neoplasia or all PE were similar (AUC = 0.6710 ± 0.04 and AUC = 0.6972 ± 0.038) (Fig. 2c and Additional file 7: Table S4.1). The best specificity and sensitivity for BDNF were ~ 86.05% and ~ 49.51% (Additional file 7: Table S4.2).

The diagnostic value of BDNF (AUC = 0.69) seems slightly lower than the one of SMRP (AUC = 0.76 to 0.87) [6], the best MPM soluble biomarker to date. However, BDNF is expressed by all subtypes of MPM unlike SMRP which is not expressed by SM [2]. Then, an association of these two biomarkers has a strong potential to improve the sensitivity and the specificity of MPM diagnosis. Comparison of BDNF diagnostic value with fibulin-3 is currently complicated due to heterogeneity in the results obtained with this biomarker [2].

### Prognostic value of BDNF in pleural effusions from patients

In several cancers, BDNF was described as overexpressed in the tumor environment [4, 7] and can be associated with poor survival [8]. Then, we evaluated the prognosis value of BDNF in MPM PE. Interestingly, as in mRNA study, patients with BDNF above median presented a significantly lower survival than the others (8.3 versus 13 months; *p* = 0.0061) (Fig. 2d and Additional file 4: Table S2). This association between high BDNF and poor survival suggests an implication of this protein in the development of the pathology.

Whereas prognostic value of SMRP remains inconclusive [2], patients with high BDNF have a shorter survival than patients with low BDNF. In PE, this observation is not related to MPM subtype. Indeed, in this cohort, SM, the most aggressive subtype of mesothelioma, only represent 7% of the cases and therefore cannot be responsible for this result. In PE, these characteristics are similar to the prognostic value of Fibulin-3 [2].

### Evaluation of BDNF on angiogenesis

**Fig. 3:**
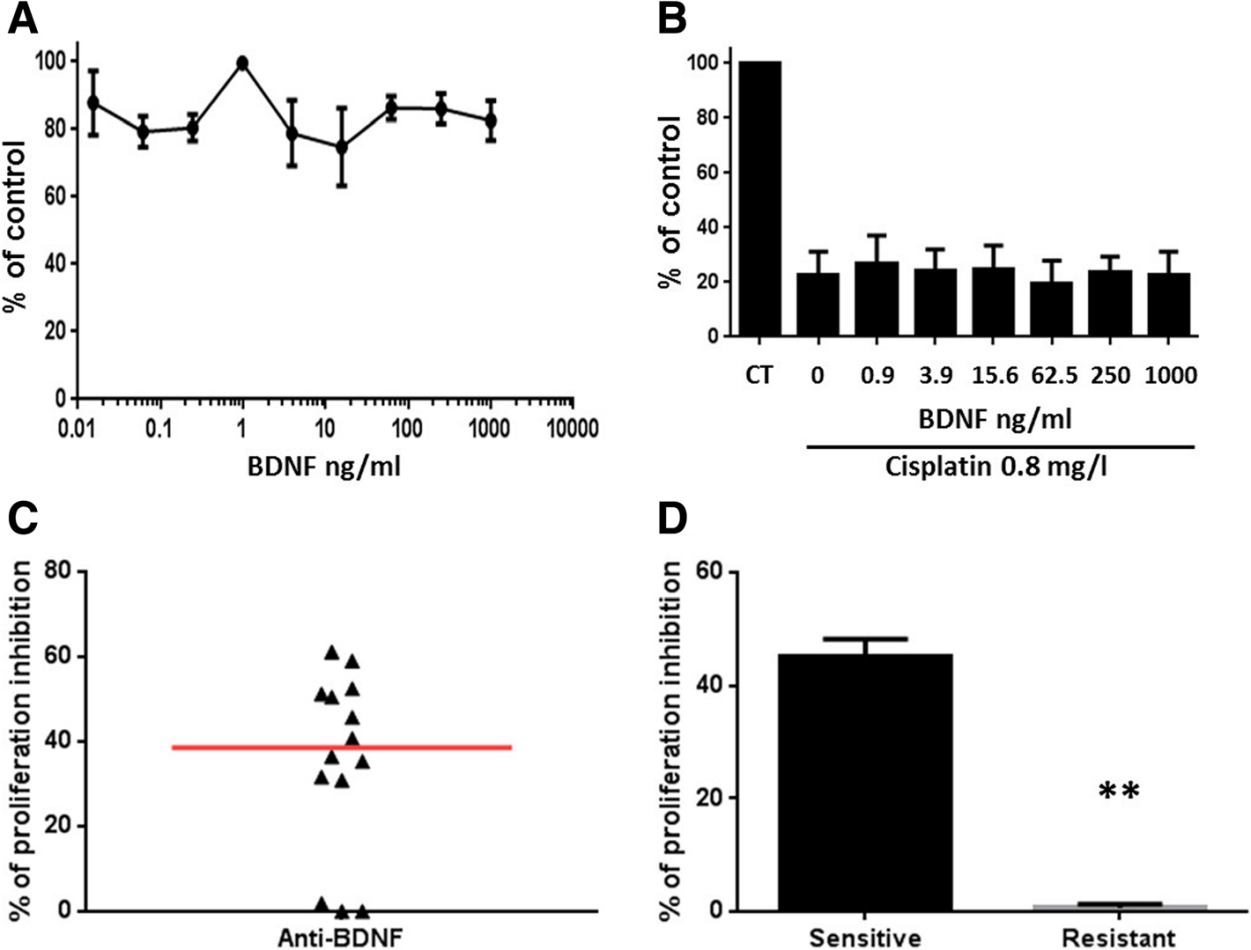
Evaluation of BDNF activity on MPM cells and on PE-induced HUVEC proliferation. **a** Effect of BDNF on MPM cell growth. **b** Effect of BDNF on cisplatin toxicity on MPM cells. **c** Effect of an anti-BDNF blocking antibody on MPM pleural effusion-induced HUVEC proliferation (*n* = 14). Red bars correspond to median. **d** Segregation of pleural effusions in sensitive (*n* = 11) and resistant (*n* = 3) groups according to the anti-BDNF blocking antibody activity

Several studies have demonstrated a pro-tumoral autocrine action of BDNF on cancer cells [8]. To evaluate this activity on MPM cells, expressions of BDNF receptors (TrkB and p75NTR) were measured first. Additional file 8: Figure S4A showed a heterogeneous and significant reduced expression of TrkB in MPM cells compared with MC. p75NTR expression was also heterogeneous in MPM cells and similar to MC (Additional file 8: Figure S4B). Figure 3a and b show that BDNF had no effect on MPM cell growth and sensitivity to cisplatin. These results suggest that BDNF has no autocrine action on MPM cells.

BDNF was also described as involved on angiogenesis in different cancer types [9]. We thus studied this property by measuring the induction of HUVEC proliferation. First, we showed that MPM PE induced angiogenesis by leading to an increase of HUVEC tube formation and proliferation (Additional file 9: Figure S5A-B). Figure 3c shows that an anti-BDNF blocking antibody (from rabbit, Abcam) reduced significantly by ~ 31% the MPM PE-induced HUVEC proliferation. A detailed analysis of the results led to the segregation of the MPM PE in a sensitive group to BDNF blocking (*n* = 11) and in a resistant group (*n* = 3) (Fig. 3d). These results were confirmed using another anti-BDNF blocking antibody (from chicken, Abcam) (Additional file 9: Figure S5C).

These observations demonstrate the strong implication of BDNF in the PE-induced angiogenesis. However, the resistance of some PE to the blocking antibody demonstrates that BDNF is not the only player participating to this process. This is also supported by the observation that the activity of the blocking antibody is not correlated to BDNF concentrations in PE (Additional file 10: Figure S6). Previous works have shown that, in some cancers, BDNF can induce expression of the vascular endothelial growth factor (VEGF), well known to induce angiogenesis, [9]. Thus, we measured VEGF in MPM PE. No evident correlation between BDNF and VEGF was observed (Additional file 11: Figure S7A). However, we did not observe samples with high BDNF and low VEGF. Moreover, in PE with BDNF higher than median value, a positive correlation with VEGF was observed (Additional file 11: Figure S7B). This suggests that VEGF can be dependent of BDNF in some PE. As observed for BDNF, the activity of the blocking antibody was not correlated to VEGF concentrations (Additional file 11: Figure S7C). These results show that VEGF cannot explain anti-angiogenic effect of the BDNF blocking antibody.

Recently, in the MAPS study, it was shown that the combination pemetrexed/cisplatin in association with bevacizumab (anti-VEGF) improves overall survival of MPM patients [10]. This clinical trial demonstrates the interest of targeting angiogenesis in MPM. Regarding our results, this suggests that BDNF could be an interesting target in MPM due to its implication in this process.

## Conclusion

Our work identifies BDNF as new interesting MPM biomarker. Moreover, due to its implication in angiogenesis, BDNF could also be a new potential therapeutic target.

## Additional files

Additional file 1: **Figure S1.** BDNF expression in MPM and lung ADCA cell lines using microarray data. **p < 0.01. (PDF 84 kb)

Additional file 2: **Table S1.1**; **S1.2** and **S1.3.** Characteristics of patients from the different biocollections. (DOCX 16 kb)

Additional file 3: **Figure S2.** BDNF expression in MPM subtypes. A) mRNA expression of *BDNF* in frozen MPM tumors samples. EM: epithelioid MPM; SM: sarcomatoid MPM; DM: desmoplastic MPM; BM: biphasic MPM. Red bars correspond to median. **p < 0.01. B) Pleural effusion BDNF values in patients with epithelioid MPM (EM), biphasic MPM (BM), sarcomatoid MPM (SM), lung ADCA, other neoplasia or BPE. Red bars correspond to median. MPM: malignant pleural mesothelioma; ADCA: adenocarcinoma; BPE: benign pleural effusion. (PDF 111 kb)

Additional file 4: **Table S2.** Survival of MPM patients with *BDNF* gene expression below and above median. (DOCX 14 kb)

Additional file 5: **Table S3.** Signification of abbreviations used in Fig. 1e. (DOCX 15 kb)

Additional file 6: **Figure S3.** BDNF expression in MPM, neoplasia and primary mesothelial cells. A) mRNA expression of BDNF in MPM neoplastic cells and primary mesothelial cells (MC). Black squares: lung ADCA cell lines; open square: pancreatic cancer cell line; black triangle: mesothelial cells from pleura; open circle: mesothelial cells from peritoneum. Red bars correspond to median. *p < 0.05; **p < 0.01. B) Cellular expression of BDNF in MPM and mesothelial cells (MESF-1). Immunofluorescence of 3 MPM and 1 primary peritoneal mesothelial cell labeled with an antibody directed against BDNF. Cell nuclei were stained using Hoechst. C) BDNF secretion by MPM and neoplastic cells. Black squares: lung ADCA cell lines; open square: pancreatic cancer cell line. *p < 0.05. (PDF 135 kb)

Additional file 7: **Tables S4.1** and **S4.2.** Diagnostic value of BDNF in pleural effusions. (DOCX 14 kb)

Additional file 8: **Figure S4.** Study of the BDNF pathway in MPM cells. mRNA expression of TrkB (A) and p75NTR (B) in MPM and primary mesothelial cells (MC). black triangle: mesothelial cells from pleura. Open circle: mesothelial cells from peritoneum. Red bars correspond to median. (PDF 97 kb)

Additional file 9: **Figure S5.** Study of angiogenesis induced by MPM pleural effusions. A) Tube formation assay. HUVEC cells were seeded on a matrix of low growth factor matrigel in EBM medium containing 2% serum. After 8 h, EBM 2% serum, EBM 10% serum (positive control) or MPM PE were added on cells for 24 h. B - C) Endothelial growth assay. HUVEC were seeded on 96-well plate at 5 × 10^4^ cells per wells. After 24 h, cells were incubated with EBM medium contaning 2% serum or MPM PE (*n* = 14) (B), or with 2 sensitive and 2 resistant MPM PE preincubated or not with a chicken anti-BDNF blocking antibody (40 μg/ml) (Abcam) for 72 h (C). Cell growth was measured using Uptiblue cell counting reagent (Interchim). (PDF 263 kb)

Additional file 10: **Figure S6.** Correlation between anti-BDNF blocking antibody activity and pleural effusion BDNF levels. (PDF 7 kb)

Additional file 11: **Figure S7.** Expression of VEGF and BDNF in pleural effusions from MPM patients (*N* = 37). A) Correlation between VEGF and BDNF levels. B) Correlation between VEGF and BDNF levels in samples with BDNF levels higher than the median value. C) Correlation between anti-BDNF blocking antibody activity and pleural effusion VEGF levels. (PDF 104 kb)

Additional file 12: Materials and Methods. (DOCX 28 kb)

## Abbreviations

ADCA: Adenocarcinoma
AUC: Area under the curve
BDNF: Brain-derived neurotrophic factor
BM: Biphasic MPM
BPE: Benign pleural effusion
CCS: Cell culture supernatant
DM: Desmoplastic MPM
ELISA: Enzyme-linked immunosorbent assay
EM: Epithelioid MPM
FCS: Fetal calf serum
HUVEC: Human umbilical vein endothelial cell
LUAD: Lung adenocarcinoma
LUSC: Lung squamous cell carcinoma
MC: Mesothelial cells
MPM: Malignant pleural mesothelioma
PCR: Polymerase chain reaction
PE: Pleural effusions
ROC: Receiver operating characteristic
RPMI: Roswell Park Memorial Institute medium
RSEM values: RNA-seq by Expectation Maximization values
SM: Sarcomatoid MPM
SMRP: Soluble mesothelin-related peptide
TCGA: The Cancer Genome Atlas
TrkB: Tropomyosin-related kinase receptors B
VEGF: Vascular endothelial growth factor

## References

[1] Vogelzang NJ, Rusthoven JJ, Symanowski J, Denham C, Kaukel E, Ruffie P (2003). Phase III study of pemetrexed in combination with cisplatin versus cisplatin alone in patients with malignant pleural mesothelioma. J Clin Oncol.

[2] Sun HH, Vaynblat A, Pass HI (2017). Diagnosis and prognosis-review of biomarkers for mesothelioma. Ann Transl Med.

[3] Gueugnon F, Leclercq S, Blanquart C, Sagan C, Cellerin L, Padieu M (2011). Identification of novel markers for the diagnosis of malignant pleural mesothelioma. Am J Pathol.

[4] Duysinx BC, Paulus A, Heinen V, Nguyen D, Henket M, Corhay JL (2011). Diagnostic value of neurotrophin expression in malignant pleural effusions. Exp Ther Med.

[5] Nockher WA, Renz H (2005). Neurotrophins in clinical diagnostics: pathophysiology and laboratory investigation. Clin Chim Acta.

[6] Pantazopoulos I, Boura P, Xanthos T, Syrigos K (2013). Effectiveness of mesothelin family proteins and osteopontin for malignant mesothelioma. Eur Respir J.

[7] Ricci A, Mariotta S, Pompili E, Mancini R, Bronzetti E, De Vitis C (2010). Neurotrophin system activation in pleural effusions. Growth Factors.

[8] Radin DP, Patel P (2017). BDNF: an oncogene or tumor suppressor?. Anticancer Res.

[9] Tajbakhsh A, Mokhtari-Zaer A, Rezaee M, Afzaljavan F, Rivandi M, Hassanian SM (2017). Therapeutic potentials of BDNF/TrkB in breast Cancer; current status and perspectives. J Cell Biochem.

[10] Zalcman G, Mazieres J, Margery J, Greillier L, Audigier-Valette C, Moro-Sibilot D (2016). Bevacizumab for newly diagnosed pleural mesothelioma in the mesothelioma Avastin cisplatin Pemetrexed study (MAPS): a randomised, controlled, open-label, phase 3 trial. Lancet.

