# Supplementary figures and images for: Brain-derived neurotrophic factor, a new soluble biomarker for malignant pleural mesothelioma involved in angiogenesis
Source: Mol Cancer. 2018 Oct 11;17:148. doi: 10.1186/s12943-018-0891-0 (PMC6180566; doi:10.1186/s12943-018-0891-0)

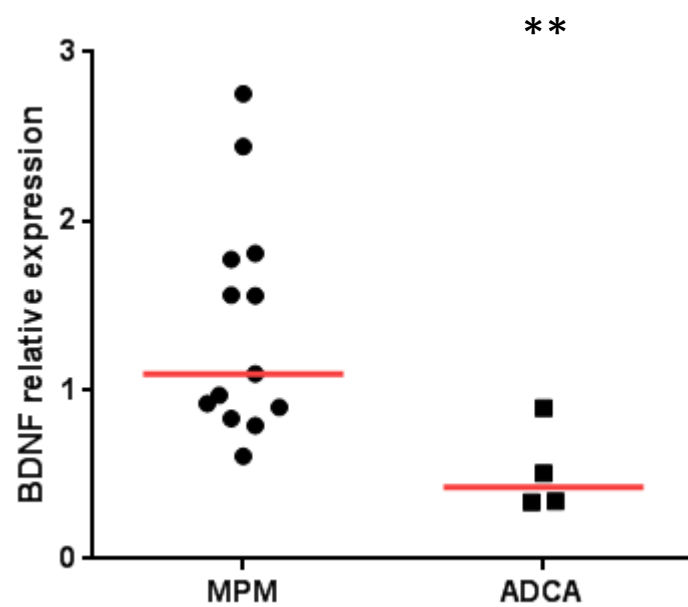

Supplement: Supplementary file 1 — Figure S1. BDNF expression in MPM and lung ADCA cell lines using microarray data. **p < 0.01. (PDF 84 kb) [file 12943_2018_891_MOESM1_ESM.pdf]

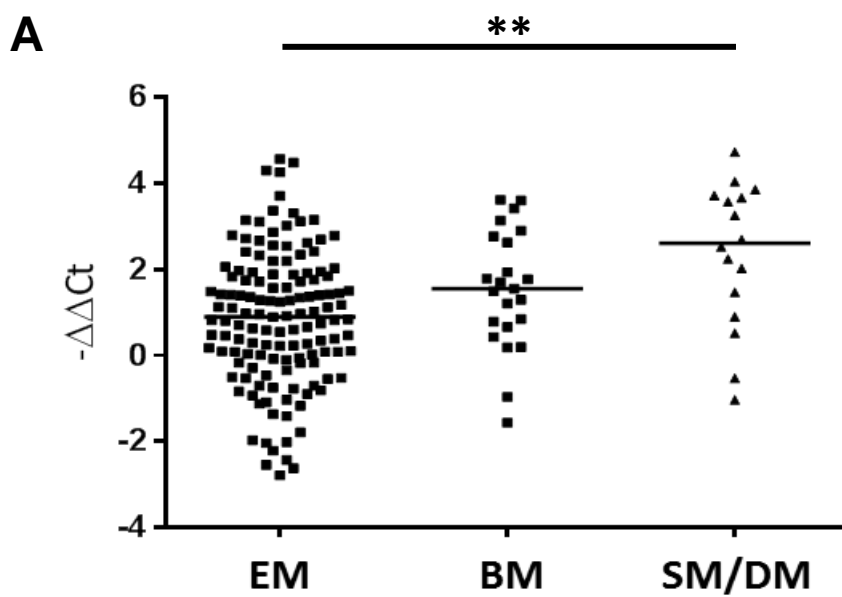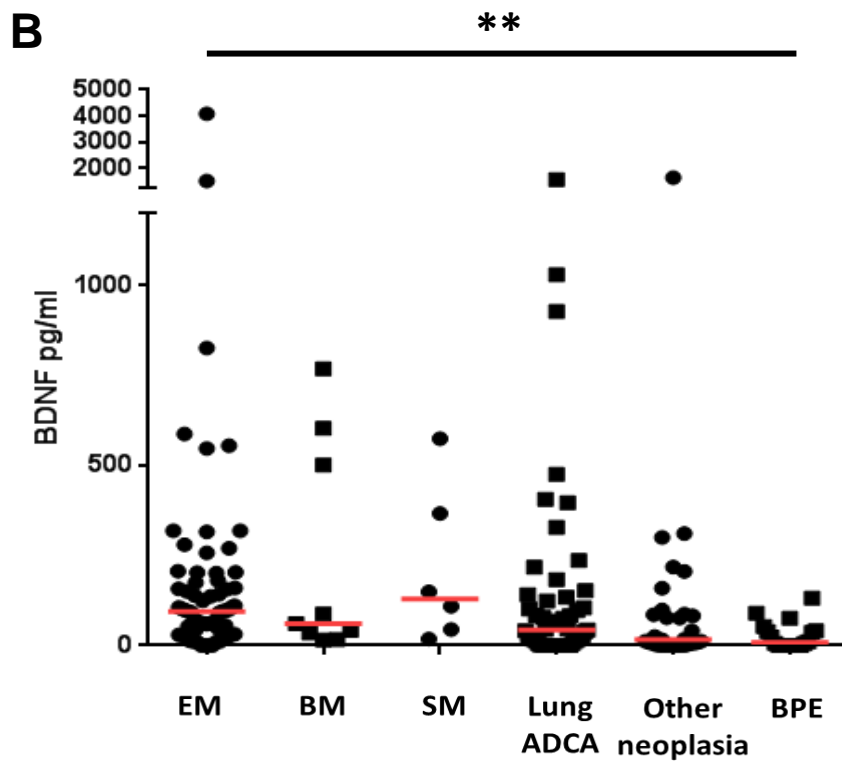

|        |        |        |        |        |        |       |
|--------|--------|--------|--------|--------|--------|-------|
| N=     | 67     | 9      | 6      | 51     | 34     | 18    |
| Median | 95.32  | 60.79  | 128.80 | 42.78  | 16.70  | 8.87  |
| Mean   | 214.10 | 237.00 | 210.40 | 146.00 | 103.60 | 28.50 |
| SD     | 530.50 | 299.10 | 217.30 | 289.60 | 281.80 | 37.80 |

Supplement: Supplementary file 3 — Figure S2. BDNF expression in MPM subtypes. A) mRNA expression of BDNF in frozen MPM tumors samples. EM: epithelioid MPM; SM: sarcomatoid MPM; DM: desmoplastic MPM; BM: biphasic MPM. Red bars correspond to median. **p < 0.01. B) Pleural effusion BDNF values in patients with epithelioid MPM (EM), biphasic MPM (BM), sarcomatoid MPM (SM), lung ADCA, other neoplasia or BPE. Red bars correspond to median. MPM: malignant pleural mesothelioma; ADCA: adenocarcinoma; BPE: benign pleural effusion. (PDF 111 kb) [file 12943_2018_891_MOESM3_ESM.pdf]

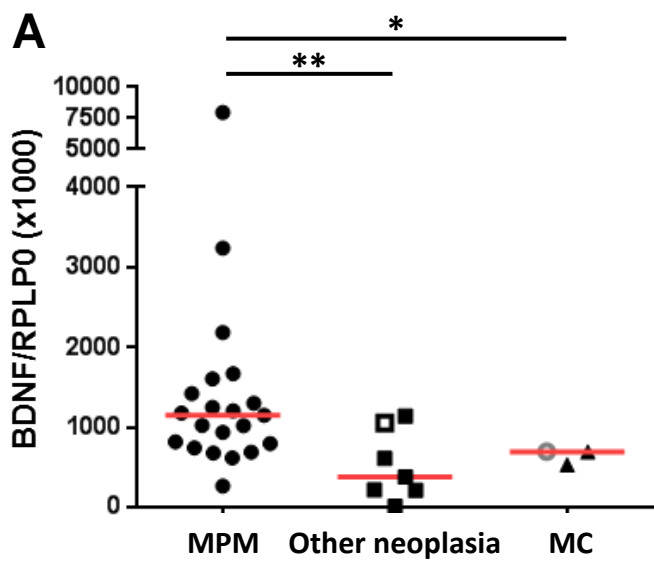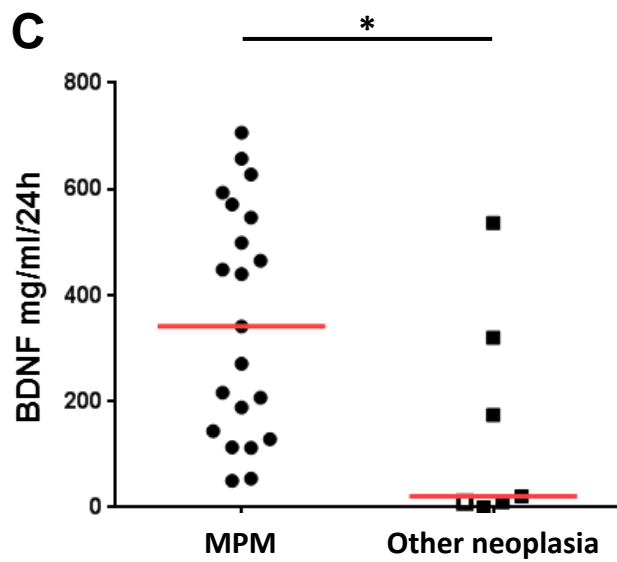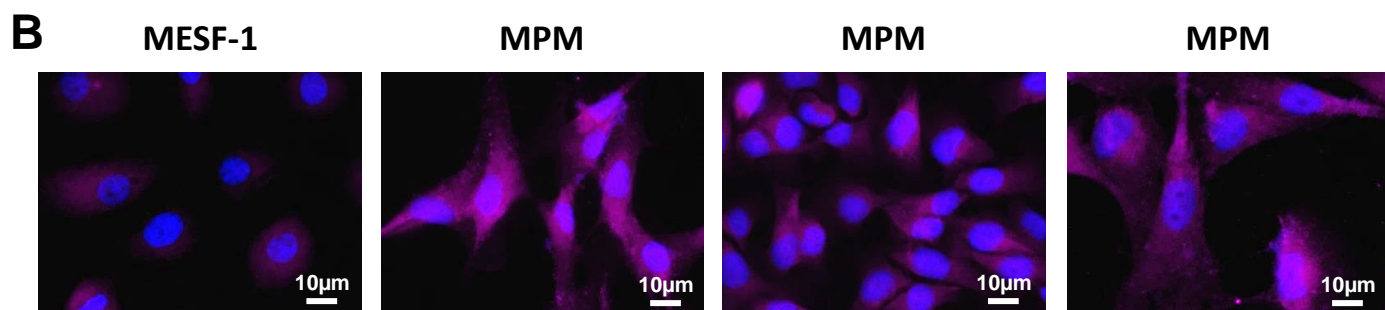

Supplement: Supplementary file 6 — Figure S3. BDNF expression in MPM, neoplasia and primary mesothelial cells. A) mRNA expression of BDNF in MPM neoplastic cells and primary mesothelial cells (MC). Black squares: lung ADCA cell lines; open square: pancreatic cancer cell line; black triangle: mesothelial cells from pleura; open circle: mesothelial cells from peritoneum. Red bars correspond to median. *p < 0.05; **p < 0.01. B) Cellular expression of BDNF in MPM and mesothelial cells (MESF-1). Immunofluorescence of 3 MPM and 1 primary peritoneal mesothelial cell labeled with an antibody directed against BDNF. Cell nuclei were stained using Hoechst. C) BDNF secretion by MPM and neoplastic cells. Black squares: lung ADCA cell lines; open square: pancreatic cancer cell line. *p < 0.05. (PDF 135 kb) [file 12943_2018_891_MOESM6_ESM.pdf]

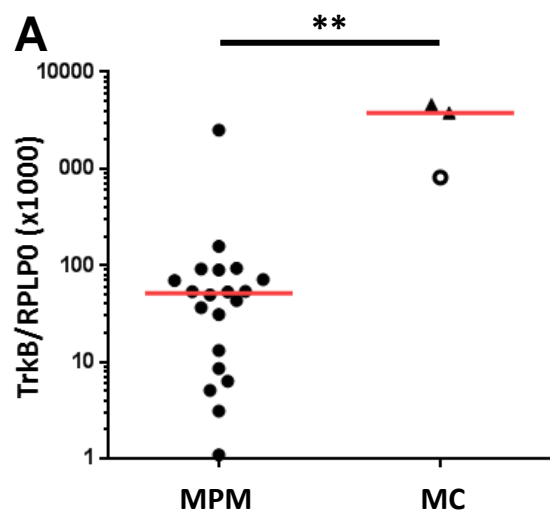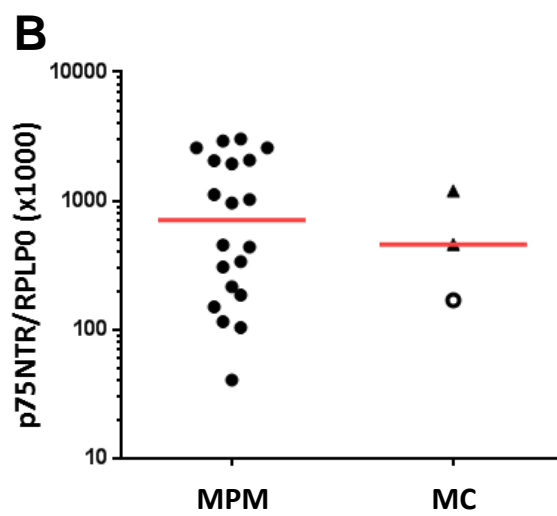

Supplement: Supplementary file 8 — Figure S4. Study of the BDNF pathway in MPM cells. mRNA expression of TrkB (A) and p75NTR (B) in MPM and primary mesothelial cells (MC). black triangle: mesothelial cells from pleura. Open circle: mesothelial cells from peritoneum. Red bars correspond to median. (PDF 97 kb) [file 12943_2018_891_MOESM8_ESM.pdf]

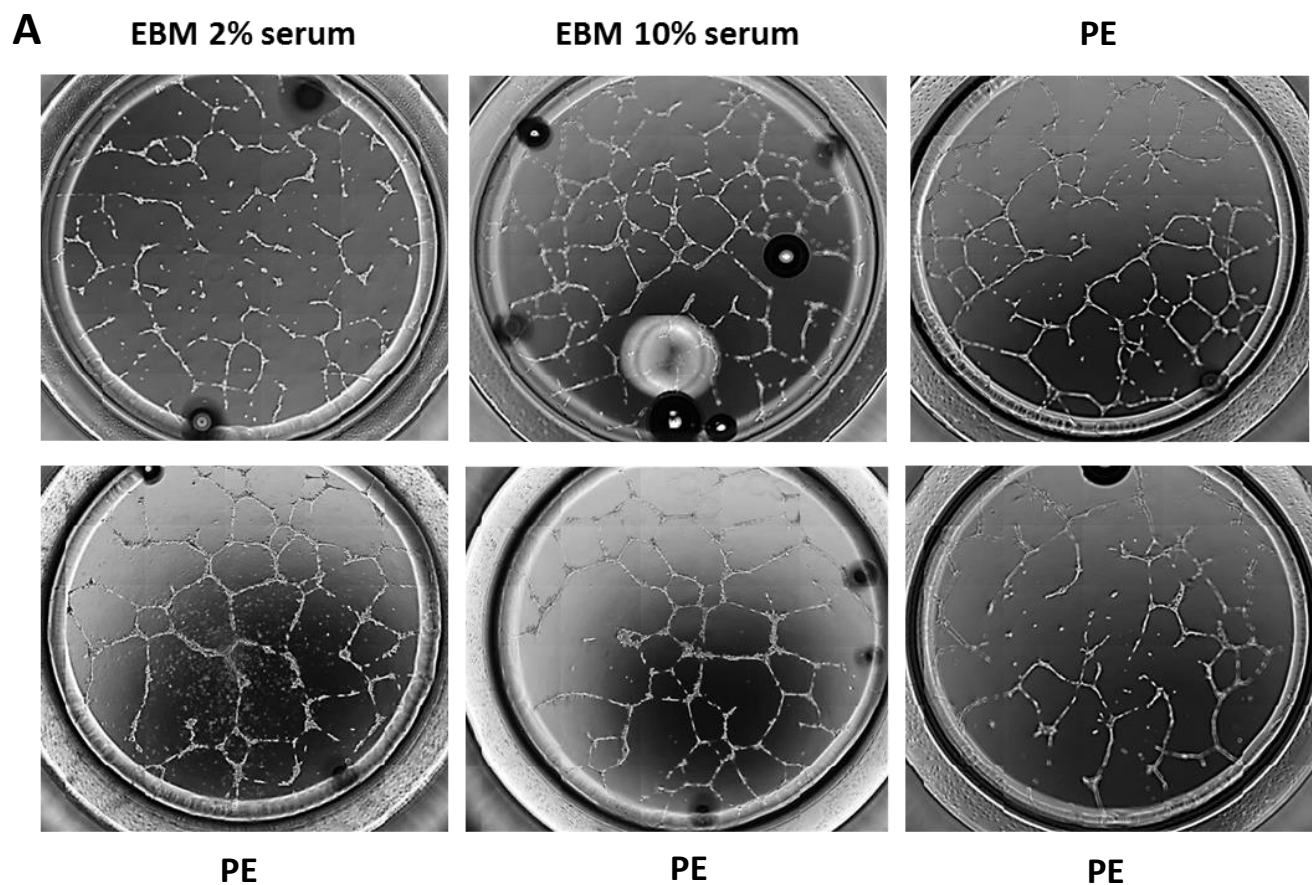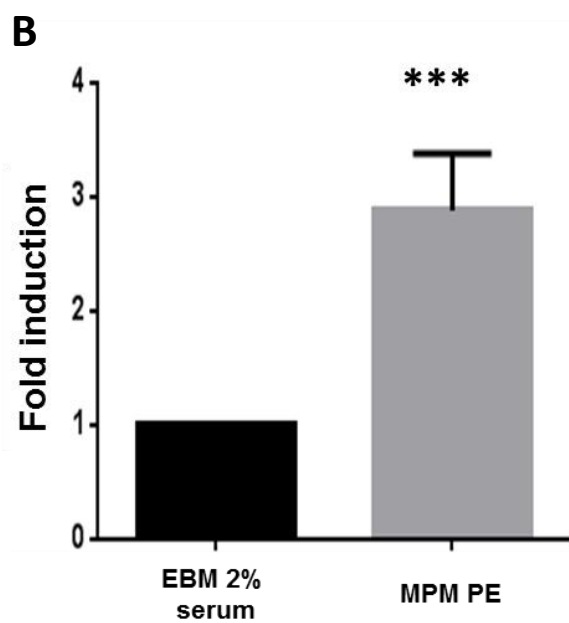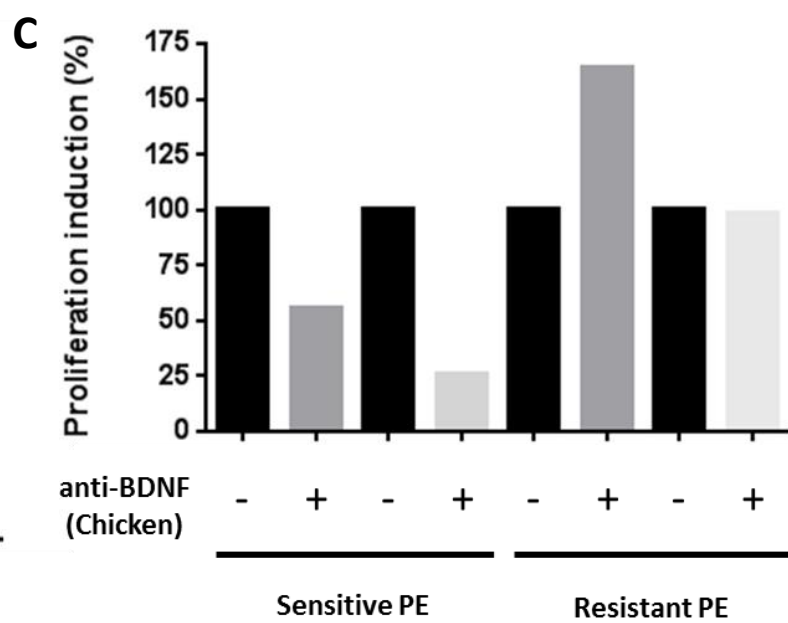

Supplement: Supplementary file 9 — Figure S5. Study of angiogenesis induced by MPM pleural effusions. A) Tube formation assay. HUVEC cells were seeded on a matrix of low growth factor matrigel in EBM medium containing 2% serum. After 8 h, EBM 2% serum, EBM 10% serum (positive control) or MPM PE were added on cells for 24 h. B - C) Endothelial growth assay. HUVEC were seeded on 96-well plate at 5 × 10^4^ cells per wells. After 24 h, cells were incubated with EBM medium contaning 2% serum or MPM PE (n = 14) (B), or with 2 sensitive and 2 resistant MPM PE preincubated or not with a chicken anti-BDNF blocking antibody (40 μg/ml) (Abcam) for 72 h (C). Cell growth was measured using Uptiblue cell counting reagent (Interchim). (PDF 263 kb) [file 12943_2018_891_MOESM9_ESM.pdf]

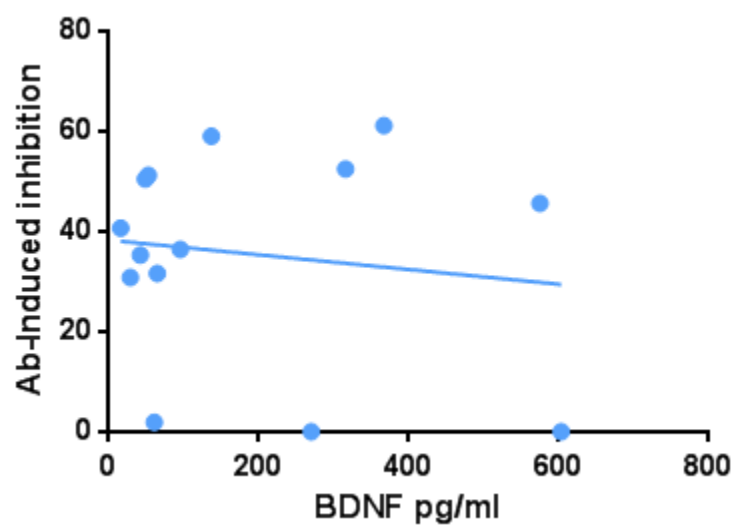

Supplement: Supplementary file 10 — Figure S6. Correlation between anti-BDNF blocking antibody activity and pleural effusion BDNF levels. (PDF 7 kb) [file 12943_2018_891_MOESM10_ESM.pdf]

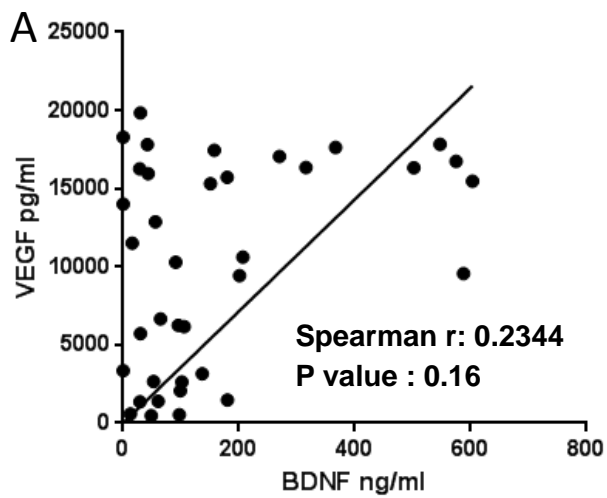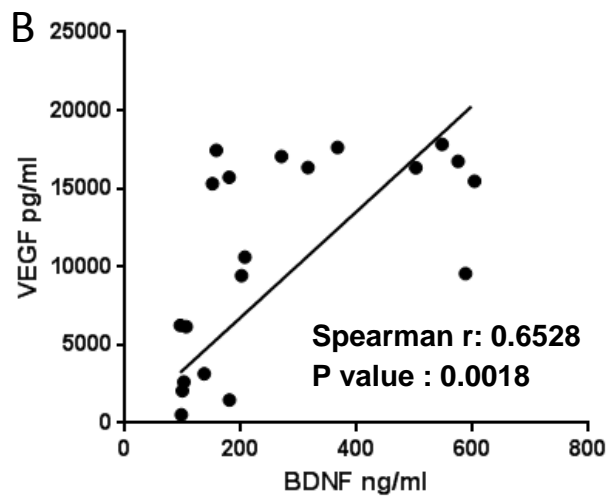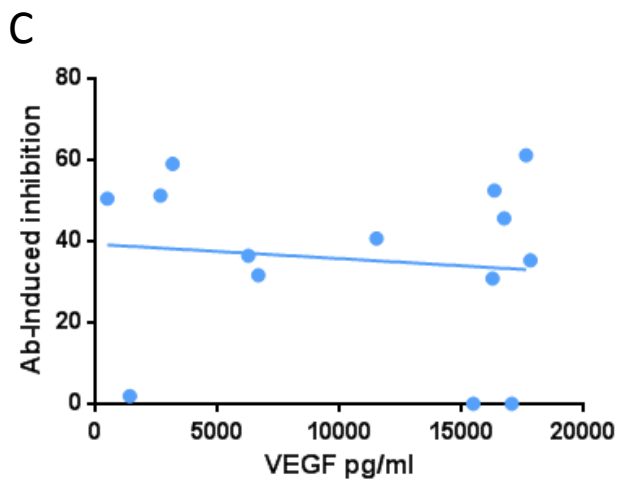

Supplement: Supplementary file 11 — Figure S7. Expression of VEGF and BDNF in pleural effusions from MPM patients (N = 37). A) Correlation between VEGF and BDNF levels. B) Correlation between VEGF and BDNF levels in samples with BDNF levels higher than the median value. C) Correlation between anti-BDNF blocking antibody activity and pleural effusion VEGF levels. (PDF 104 kb) [file 12943_2018_891_MOESM11_ESM.pdf]
