# Supplementary material for: Brain-derived neurotrophic factor, a new soluble biomarker for malignant pleural mesothelioma involved in angiogenesis
Source: Mol Cancer. 2018 Oct 11;17:148. doi: 10.1186/s12943-018-0891-0 (PMC6180566; doi:10.1186/s12943-018-0891-0)
Supplement: Supplementary file 2 — Table S1.1; S1.2 and S1.3. Characteristics of patients from the different biocollections. (DOCX 16 kb) [file 12943_2018_891_MOESM2_ESM.docx]

Table S1.1. Characteristics of patients of frozen samples

|  | **MPM** | **Normal pleura** |
| --- | --- | --- |
| Description | 179  135 epithelioid  13 sarcomatoid  23 biphasic  3 desmoplastic  1 lymphohistiocytoid  4 unspecified | 26  13 lung cancer  1 mediastinal lymphoma  1 malignant solitary fibrous tumor  1 small cell neuroendocrine carcinoma  3 pulmonary emphysema  4 pleurisy  1 spontaneous pneumothorax  2 unspecified |
| Age, y (mean ± SD) | 67.4 ± 10.5 | 56.3 ± 16.4 |
| Male sex, (%) | 76.0 | 92.0 |
| Confirmed asbestos exposure (%) | 71.5 | NA |
| MPM, malignant pleural mesothelioma | | |

Table S1.2. Characteristics of MPM patients included in TCGA dataset

|  | **MPM** | **LUAD** | | **LUSC** |
| --- | --- | --- | --- | --- |
| Description | 72  46 epithelioid  1 sarcomatoid  20 biphasic  5 diffuse | | 149  107 lung adenocarcinoma- not otherwise specified  28 lung adenocarcinoma mixed subtype  6 lung papillary adenocarcinoma  4 lung bronchioloalveolar carcinoma nonmucinous  2 lung acinar adenocarcinoma  1 mucinous (colloid) carcinoma  1 lung signet ring adenocarcinoma | 180  172 lung squamous cell carcinoma- not otherwise specified  4 lung basaloid squamous cell carcinoma  3 lung papillary squamous cell caricnoma  1 lung small cell squamous cell carcinoma |
| Age, y (mean ± SD) | 63.57 ± 9.89 | 65.32 ± 10.79 | | 68.22 ± 8.20 |
| Male sex, (%) | 83.33 | 45.64 | | 76.67 |
| Confirmed asbestos exposure (%) | 66.67 | - | | - |
| MPM, Malignant pleural mesothelioma; LUAD, lung adenocarcinoma ; LUSC, lung squamous cell carcinoma | | | | |

Table S1.3. Characteristics of patients for pleural effusion study

|  | **MPM** | **Other neoplasia** | **BPE** |
| --- | --- | --- | --- |
| Description | 86  67 epithelioid  6 sarcomatoid  9 biphasic  4 unspecified | 85  51 Lung  34 others | 18 |
| Age, y (mean ± SD) | 68.5 ± 9.7 | 64.2 ± 11.9 | 71.1 ± 11.4 |
| Male sex, (%) | 84.8 | 56.5 | 77.7 |
| Confirmed asbestos exposure (%) | 68.6 | 17.6 | 33.3 |
| MPM, malignant pleural mesothelioma ; BPE, benign pleural effusion | | | |
