# Supplementary material for: Brain-derived neurotrophic factor, a new soluble biomarker for malignant pleural mesothelioma involved in angiogenesis
Source: Mol Cancer. 2018 Oct 11;17:148. doi: 10.1186/s12943-018-0891-0 (PMC6180566; doi:10.1186/s12943-018-0891-0)
Supplement: Supplementary file 4 — Table S2. Survival of MPM patients with BDNF gene expression below and above median. (DOCX 14 kb) [file 12943_2018_891_MOESM4_ESM.docx]

Table S2. Survival of MPM patients with *BDNF* gene expression below and above median

| Survival (months) | **Frozen tumors cohort (mRNA)** | **TCGA database**  **(mRNA)** | **Pleural effusion cohort (protein)** |
| --- | --- | --- | --- |
| BDNF below median  BDNF above median | 21.1  15.9 | 27.5  12.4 | 13.0  8.3 |
| *p* | 0.0736* | <0.0001 | 0.0061 |

* *p*=0.0401 at 3 years
