# Supplementary material for: Brain-derived neurotrophic factor, a new soluble biomarker for malignant pleural mesothelioma involved in angiogenesis
Source: Mol Cancer. 2018 Oct 11;17:148. doi: 10.1186/s12943-018-0891-0 (PMC6180566; doi:10.1186/s12943-018-0891-0)
Supplement: Supplementary file 5 — Table S3. Signification of abbreviations used in Fig. 1e. (DOCX 15 kb) [file 12943_2018_891_MOESM5_ESM.docx]

Table S3: Signification of abbreviations used in figure 1e

| **Abbreviations** | **Significations** |
| --- | --- |
| ACC | Adrenocortical carcinoma |
| BLCA | Bladder Urothelial Carcinoma |
| BRCA | Breast invasive carcinoma |
| CESC | Cervical squamous cell carcinoma and endocervical adenocarcinoma |
| CHOL | Cholangiocarcinoma |
| COAD | Colon adenocarcinoma |
| ESCA | Esophageal carcinoma |
| GBM | Glioblastoma multiform |
| HNSC | Head and Neck squamous cell carcinoma |
| KICH | Kidney Chromophobe |
| KIRC | Kidney renal clear cell carcinoma |
| KIRP | Kidney renal papillary cell carcinoma |
| LAML | Acute Myeloid Leukemia |
| LIHC | Liver hepatocellular carcinoma |
| LGG | Brain Lower Grade Glioma |
| LUAD | Lung adenocarcinoma |
| LUSC | Lung squamous cell carcinoma |
| DLBC | Lymphoid Neoplasm Diffuse Large B-cell Lymphoma |
| MESO | Mesothelioma |
| OV | Ovarian serous cystadenocarcinoma |
| PAAD | Pancreatic adenocarcinoma |
| PCPG | Pheochromocytoma and Paraganglioma |
| PRAD | Prostate adenocarcinoma |
| READ | Rectum adenocarcinoma |
| SARC | Sarcoma |
| SKCM | Skin Cutaneous Melanoma |
| STAD | Stomach adenocarcinoma |
| TGCT | Testicular Germ Cell Tumors |
| THCA | Thyroid carcinoma |
| THYM | Thymoma |
| UCS | Uterine Carcinosarcoma |
| UCEC | Uterine Corpus Endometrial Carcinoma |
| UVM | Uveal Melanoma |
