# Supplementary material for: Brain-derived neurotrophic factor, a new soluble biomarker for malignant pleural mesothelioma involved in angiogenesis
Source: Mol Cancer. 2018 Oct 11;17:148. doi: 10.1186/s12943-018-0891-0 (PMC6180566; doi:10.1186/s12943-018-0891-0)
Supplement: Supplementary file 7 — Tables S4.1 and S4.2. Diagnostic value of BDNF in pleural effusions. (DOCX 14 kb) [file 12943_2018_891_MOESM7_ESM.docx]

Table S4.1. ROC Curve data for ability of BDNF to differentiate MPM from other neoplasia and/or BPE in pleural effusions

|  | **AUC** | **95% Confidence Interval** | **SE** | **p** |
| --- | --- | --- | --- | --- |
| MPM vs all effusions | 0.6972 | 0.6223 to 0.7721 | 0.038 | <0.0001 |
| MPM vs other neoplasia | 0.6710 | 0.5891 to 0.7529 | 0.041 | 0.0001 |
| ROC, receiver operating characteristic; AUC, area under the curve; SE, standard error; MPM, malignant pleural mesothelioma; BPE, benign pleural effusion | | | | |

Table S4.2. Theoritical Best Cut-off values to differentiate MPM from other neoplasia and/or BPE in pleural effusions

|  | **Cutoff**  **pg/ml** | **Specificity %** | **Sensitivity %** |
| --- | --- | --- | --- |
| MPM vs all effusions | 20.16 | 86 .05 | 49.51 |
| MPM vs other neoplasia | 20.16 | 86.05 | 48.24 |
| MPM, malignant pleural mesothelioma | | | |
