# Supplementary material for: Brain-derived neurotrophic factor, a new soluble biomarker for malignant pleural mesothelioma involved in angiogenesis
Source: Mol Cancer. 2018 Oct 11;17:148. doi: 10.1186/s12943-018-0891-0 (PMC6180566; doi:10.1186/s12943-018-0891-0)
Supplement: Supplementary file 12 — Materials and Methods. (DOCX 28 kb) [file 12943_2018_891_MOESM12_ESM.docx]

**Brain-derived neurotrophic factor (BDNF), a new soluble biomarker for malignant pleural mesothelioma involved in angiogenesis**

**Supplementary Informations**

**Materials & methods**

**Additional material legends**

**Tables**

**MATERIALS AND METHODS**

**Collection of MPM frozen tumors samples**

Frozen MPM tumor samples were retrieved from the French Mesobank collection (biobanks of two French hospitals: CHRU de Lille and CHRU de Nice) and the biobank collection of HEGP (Hôpital Européen Georges Pompidou) in Paris. Patients were diagnosed from 2003 to 2016 and certified by Mesopath as MPM. Normal pleura were obtained after stripping and dissecting the parietal pleura from patients with lung cancer, mediastinal lymphoma, malignant solitary fibrous tumor, small cell neuroendocrine carcinoma, pulmonary emphysema, pleurisy or spontaneous pneumothorax. The percentage of tumor cells in MPM samples was estimated by histologic examination. All patients gave signed informed consent. All the collected samples and the associated clinical information were registered in a database (DC-2013-1963) validated by the French research ministry.

**RNA isolation and Taqman PCR from frozen normal and tumor tissues.**

Total RNA was isolated using Trizol (Thermo Fisher Scientific) or AllPrep DNA/RNA/miRNA Universal kit (Qiagen) according to the manufacturer’s protocol. 1.5µg of total RNA was reverse-transcribed using High-Capacity cDNA Reverse Transcription Kit (Thermo Fisher Scientific). Real-time PCR (RT-PCR) was carried out using ABI Prism 7900HT Real-Time PCR System. PCR reactions were performed using Taqman assays (Thermo Fisher Scientific) according to the manufacturer’s instructions. *BDNF* transcript level was normalized by the mean of the expression values of the five housekeeping genes Ribosomal *18S*, *ACTB*, *CLTC*, *GAPDH* and *TBP* (ΔCt) and then normalized by the mean of 26 normal pleura samples (-ΔΔCt). The following Taqman assays have been used: *BDNF* (Hs02718934_s1), *18S* (Hs03928990_g1), *ACTB* (Hs01060665_g1), *CLTC* (Hs00964504_m1), *GAPDH* (Hs02758991_g1) and *TBP* (Hs00427620_m1).

**Analysis of The Cancer Genome Atlas (TCGA) dataset**

All RNAseqv2 samples from the TCGA-MESO dataset (n=72 patients), TCGA-LUAD (n=149 patients) and TCGA-LUSC (n=180 patients) are available on the Broad’s Genome Data Analysis Center (http://gdac.broadinstitute.org/). Gene expressions as RNA-seq by Expectation Maximization values (RSEM values) were analyzed. Clinical data for these samples were downloaded from firebrowse (http://firebrowse.org ; version 2016_01_28 for MESO, LUAD and LUSC).

**Collection of pleural effusions and mesothelioma cell lines**

Pleural-effusions from patients with a suspicion of mesothelioma were aseptically collected by thoracocentesis at the Laënnec Hospital (St-Herblain, France) between 1998 and 2016. Samples were centrifuged at 1000 g in a Heraeus Multifuge for 20 minutes at +4°C and supernatants were aliquoted and stored at −80°C. Diagnoses were established by both fluid cytology and immunohistochemical staining of pleural biopsies performed by our pathology department, Laënnec Hospital (St-Herblain, France), then externally confirmed by Mesopath, the French panel of pathology experts for the diagnosis of mesothelioma. All recruited patients had received no prior anticancer therapy and gave signed, informed consent. All the collected samples and the associated clinical information were registered in a database (DC-2011-1399) validated by the French research ministry.

The mesothelioma and other neoplasia cell lines were established from pleural fluids of patients in our laboratory and they can therefore not be authenticated [[19](#_ENREF_19)]. Isolation and culture of normal mesothelial cells were described previously [[19](#_ENREF_19)]. All cell lines were maintained in RPMI medium (Invitrogen) supplemented with 2 mM L-glutamine, 100 IU/ml penicillin, 0.1 mg/ml Streptomycin and 10% heat-inactivated fetal calf serum (FCS) (Eurobio) and cultured at 37°C in a 5% CO_2_ atmosphere. The primary peritoneal mesothelial cells, MES-F, were purchased from Tebu-bio biosciences and cultured according to the manufacturer’s recommendations.

**Enzyme-Linked Immunosorbent Assays**

BDNF titrations were performed with the Human BDNF DuoSet (R&D Systems, DY248) following the manufacturers’ recommendations. VEGF levels were measured using Human VEGF quantikine ELISA kit (R&D Systems).

**RNA isolation and real-time RT-PCR from cell lines.**

Total RNA was isolated using the Nucleospin® RNAII Kit according to the manufacturer’s protocol (Macherey-Nagel). One microgram of total RNA was reverse-transcribed using Moloney murine leukemia virus reverse transcriptase (Invitrogen). Real-time PCR (RT-PCR) was carried out using an Mx3500P thermocycler (Stratagene). PCR reactions were performed using QuantiTect Primer Assays (Qiagen) and the RT² Real-Time SYBR-Green/ROX PCR Mastermix (Thermofischer Scientific), according to the manufacturer’s instructions. The relative amount of the target RNA was determined using the Mx4000 software, by comparison with the corresponding standard curve for each sample performed in duplicate. Each transcript level was normalized by division with the expression values of the acidic ribosomal phosphoprotein P0 housekeeping gene (*RPLP0*), used as an internal standard.

**Immunofluorescence**

Cells were seeded in µ-slide 8 wells (Ibidi) at a density of 1.5x10^4^cells/well. 24h later, cells were washed twice with PBS and fixed with 4% paraformaldehyde in PBS, 15min at room temperature. Cells were permeabilized with 0.05% Triton X-100 (Merck) /0.05% Tween-20 (Sigma-Aldrich) in PBS (5min) and incubated with anti-BDNF antibody at 1µg/ml (Abcam, ab6201) in PBS/1% BSA for 1h. Cells were washed twice with PBS and incubated with an Alexa Fluor 647 conjugated secondary antibody (Molecular Probes, A21245) for 1h. After an additional PBS wash, cell nuclei were stained with 1µg/ml Hoechst (Sigma-Aldrich) (5min). Fluorescence was visualized using the Axiovert200M microscopy system (Zeiss) with ApoTome module (X63 and numerial aperture 1.4).

**Preparation of cell culture supernatants**

Cell culture supernatants were collected as follows: 2 × 10^6^ cells were plated by well in 6-well plates. After 24 hours, three rinses were performed with complete, fetal calf serum (FCS)-free Roswell Park Memorial Institute 1640 medium (Gibco). Two milliliters of complete Roswell Park Memorial Institute 1640 medium containing 2% FCS were added to each well and culture supernatants were collected 24 hours later, centrifuged at 1000 g for 10 minutes, aliquoted and stored at −80°C.

**MPM cell growth**

Cell growth was monitored using Uptiblue cell counting reagent (Interchim) as previously described [[20](#_ENREF_20)]. MPM cells were seeded in 96-well plates at a density of 5x10^3^cells/well in culture medium for 24h. Then, compounds were added for an additional 72h and Uptiblue reagent (5%, v/v) was then added to the culture medium for 2h at 37°C. Fluorescence was measured at 605nm after a green epi illumination excitation using a ChemiDoc™ MP imaging system (Biorad). Quantification was performed using ImageJ 1.41o software.

**Angiogenesis evaluation**

HUVEC cells (Promocell) were seeded in 96-well plates at a density of 5x10^3^cells/well in culture medium for 24h. Then, pleural effusions were pre-incubated or not with anti-BDNF blocking antibody (Abcam, polyclonal from rabbit ab6201, 20µg/ml or polyclonal from chicken ab27932, 40µg/ml) for 30 minutes at room temperature and added at 10% on HUVEC cells. After 72h, cell growth was evaluated using Uptiblue cell counting reagent as described above.

**Statistical Analyses**

Comparisons were performed using non parametric Mann-Whitney U test or Kruskal–Wallis test followed by the Dunn’s post-hoc test. Statistical analyses, measurement of the areas under receiver operating characteristic (ROC) curves and survival studies (Log-rank Mantel-Cox test) were performed using GraphPad Prism (Prism 6 for Windows). The best theoretical cutoffs were calculated by minimizing the distance between the point with specificity=1 and sensitivity=1 and the points on the ROC curves.
